# Racial Disparities and Sex Differences in Early- and Late-Onset Colorectal Cancer Incidence, 2001–2018

**DOI:** 10.3389/fonc.2021.734998

**Published:** 2021-09-09

**Authors:** Jessica L. Petrick, Lauren E. Barber, Shaneda Warren Andersen, Andrea A. Florio, Julie R. Palmer, Lynn Rosenberg

**Affiliations:** ^1^Slone Epidemiology Center at Boston University, Boston, MA, United States; ^2^Department of Epidemiology, Boston University School of Public Health, Boston, MA, United States; ^3^Department of Population Health Sciences, University of Wisconsin School of Medicine and Public Health, Madison, WI, United States; ^4^Cancer Prevention and Control, University of Wisconsin Carbone Cancer Center, Madison, WI, United States; ^5^Department of Nutrition, Harvard T.H. Chan School of Public Health, Boston, MA, United States

**Keywords:** early-onset colorectal cancer, joinpoint analysis, National Program of Cancer Registries (NPCR), neuroendocrine tumors, racial disparities in cancer, Surveillance, Epidemiology, and End Results (SEER) program

## Abstract

**Background:**

Colorectal cancer (CRC) incidence rates have increased in younger individuals worldwide. We examined the most recent early- and late-onset CRC rates for the US.

**Methods:**

Age-standardized incidence rates (ASIR, per 100,000) of CRC were calculated using the US Cancer Statistics Database’s high-quality population-based cancer registry data from the entire US population. Results were cross-classified by age (20-49 [early-onset] and 50-74 years [late-onset]), race/ethnicity (non-Hispanic White, non-Hispanic Black, Hispanic, American Indian/Alaskan Native, Asian/Pacific Islander), sex, anatomic location (proximal, distal, rectal), and histology (adenocarcinoma, neuroendocrine).

**Results:**

During 2001 through 2018, early-onset CRC rates significantly increased among American Indians/Alaskan Natives, Hispanics, and Whites. Compared to Whites, early-onset CRC rates are now 21% higher in American Indians/Alaskan Natives and 6% higher in Blacks. Rates of early-onset colorectal neuroendocrine tumors have increased in Whites, Blacks, and Hispanics; early-onset colorectal neuroendocrine tumor rates are 2-times higher in Blacks compared to Whites. Late-onset colorectal adenocarcinoma rates are decreasing, while late-onset colorectal neuroendocrine tumor rates are increasing, in all racial/ethnic groups. Late-onset CRC rates remain 29% higher in Blacks and 15% higher in American Indians/Alaskan Natives compared to Whites. Overall, CRC incidence was higher in men than women, but incidence of early-onset distal colon cancer was higher in women.

**Conclusions:**

The early-onset CRC disparity between Blacks and Whites has decreased, due to increasing rates in Whites—rates in Blacks have remained stable. However, rates of colorectal neuroendocrine tumors are increasing in Blacks. Blacks and American Indians/Alaskan Natives have the highest rates of both early- and late-onset CRC.

**Impact:**

Ongoing prevention efforts must ensure access to and uptake of CRC screening for Blacks and American Indians/Alaskan Natives.

## Introduction

Colorectal cancer (CRC) is the third most commonly occurring cancer in the United States (US) and the third leading cause of cancer mortality (1). CRC incidence rates have decreased since the mid-1980s, but this trend changed recently due to increasing rates of early-onset colorectal cancer (EOCRC), defined as CRC arising in individuals prior to age 50 (2). EOCRC rates have also increased worldwide, particularly in high-income countries (3). Recent CRC rates in 45-year-olds are similar to rates observed in 50-year-olds prior to the advent of routine CRC screening (1), which the US Preventive Services Task Force (USPSTF) first recommended in 1996 (4). Thus, the American Cancer Society (ACS) and the USPSTF have recently updated their guidelines to recommend screening beginning at age 45 (5, 6).

Established risk factors for late-onset CRC, including obesity (7–9), physical inactivity (10), smoking (8, 11), alcohol (12), and diet (12), have also been associated with EOCRC risk in some studies. However, known CRC risk factors do not fully explain the increasing rates of EOCRC (13), indicating there are likely undiscovered risk factors for EOCRC.

Non-Hispanic Black (NHB) Americans have had the highest incidence of CRC in the US since the 1990s, including EOCRC (1). NHBs and women of any racial/ethnic group are most likely to be diagnosed with tumors in the proximal colon, where detection—especially with sigmoidoscopy—is less likely (14–16). A recent study reported that rates of EOCRC have been increasing in both non-Hispanic White (NHW) and NHB Americans (17). However, the majority of studies to date have utilized data from Surveillance, Epidemiology, and End Results (SEER) Program, which only captures data from approximately 35% of the US population. The most recent rates of EOCRC by race/ethnicity, anatomic location, and histology for the entire US population have not been reported. In the present study, we examined US CRC rates from 2001-2018 by age, sex, race/ethnicity, anatomic location, and histology. The trends of EOCRC by these factors may provide clues as to the evolving etiology of EOCRC.

## Methods

### Colorectal Cancer Data

We examined CRC incidence data from US Cancer Statistics, which includes data from the Centers for Disease Control and Prevention’s National Program of Cancer Registries (NPCR) and the National Cancer Institute’s SEER Program, spanning the years 2001 through 2018 (all available years) (18). The US Cancer Statistics database includes high-quality population-based cancer registry data from the entire US population, including all 50 states and the District of Columbia.

Incident primary CRC was defined by International Classification of Diseases for Oncology, Third Edition. Proximal colon cancer included cecum (C18.0), ascending colon (C18.2), hepatic flexure (C18.3), transverse colon (C18.4), and splenic flexure (C18.5). Distal colon cancer included descending colon (C18.6) and sigmoid (C18.7). Rectal cancer included rectum (C20.9) and rectosigmoid junction (C19.9). Overall CRC included these three anatomic locations in addition to overlapping lesions of the colon (C18.8), colon not otherwise specified (C18.9), and intestine not otherwise specified (C26.0). Colorectal adenocarcinomas were defined by ICD-O-3 histology codes (19, 20) 8140-8147, 8201, 8210-8213, 8220-8221, 8255, 8260-8265, 8310, 8323, 8331-8332, 8380, 8430, 8440, 8480-8481, 8490, 8550-8551, and 8570-8573. Colorectal neuroendocrine tumors were defined by histology codes 8013, 8240-8246, and 8249. EOCRC was defined as arising in adults 20-49 years of age, while late-onset CRC was defined as arising in persons 50-74 years of age (as the USPSTF only recommends selective screening after age 75) (6).

### Statistical Analysis

Age‐standardized incidence rates (ASIRs) and 95% confidence intervals (CIs) per 100,000 person‐years were calculated for overall CRC, proximal colon, distal colon, and rectal cancer by age (20-49 and 50-74 years), sex, and race/ethnicity (non-Hispanic White, NHW; non-Hispanic Black, NHB; Hispanic; non-Hispanic American Indian/Alaska Native, AIAN; non-Hispanic Asian/Pacific Islander, API). We also examined overall CRC rates by histology (adenocarcinoma, neuroendocrine), age, and race/ethnicity. Rates were age-adjusted to the 2000 US standard population. Corresponding standard errors and 95% CIs were calculated (21). For 2001 through 2018, ASIRs were plotted on a semi-logarithmic scale to facilitate comparison of current rates and temporal trends (22).

To examine changes in ASIRs over time by anatomic location, age, and race/ethnicity, 2-year groupings (i.e., 2001-2002 and 2017-2018) were used. Joinpoint regression models were used to test whether a change in trend was statistically significant, using the Monte Carlo Permutation method (Joinpoint Regression Program, v4.8.0.1, Information Management Services, Inc., Calverton, MD). The joinpoint regression tested a linear model with no joinpoints and assessed if more joinpoints should be added based on statistical significance. In the models, we used a maximum of two joinpoints with a minimum of four years of data between joinpoints. The annual percent change (APC) and average annual percent change (AAPC) were calculated using the natural log-transformed rates based on each 2-year period (23). The 95% CIs were calculated using the parametric method.

To examine racial/ethnic disparities and sex differences in incidence rates, incidence rate ratios (IRR=ASIR_1_/ASIR_2_) and 95% CIs were calculated. Each racial/ethnic group was compared to NHWs; men were compared to women. The IRR is a measure of relative difference; a value of 1.0 corresponds to no difference in the rates.

### Sensitivity Analysis

As the ACS and the USPSTF have updated their guidelines to recommend screening beginning at age 45 (5, 6), we conducted a sensitivity analysis to examine EOCRC trends in individuals less than 45 years of age. We also present data on CRC rates for all ages, <50 years, and ≥50 years.

## Results

The US Cancer Statistics database includes 2,585,621 CRC cases: 1,985,054 NHW, 298,735 NHB, 186,521 Hispanic, 14,806 AIAN, 83,820 API, and 16,685 Other/Unknown. For the main analyses, we excluded the CRC cases of Other/Unknown race/ethnicity.

Between 2001 to 2018, CRC rates among all ages decreased in all racial/ethnic groups and both sexes (**Supplemental Figure S1** and **Supplemental Table S1**). The overall CRC rates in NHWs decreased by 3.18% per year until 2011-2012, when the decrease in rates slowed to 1.80% per year; in NHBs, CRC rates decreased by 2.70% per year. In 2017-2018, CRC rates were 16% higher in NHBs and 6% higher in AIANs than in NHWs, while CRC rates were 10% lower in Hispanics and 20% lower in APIs compared to NHWs.

**Figure 1:**
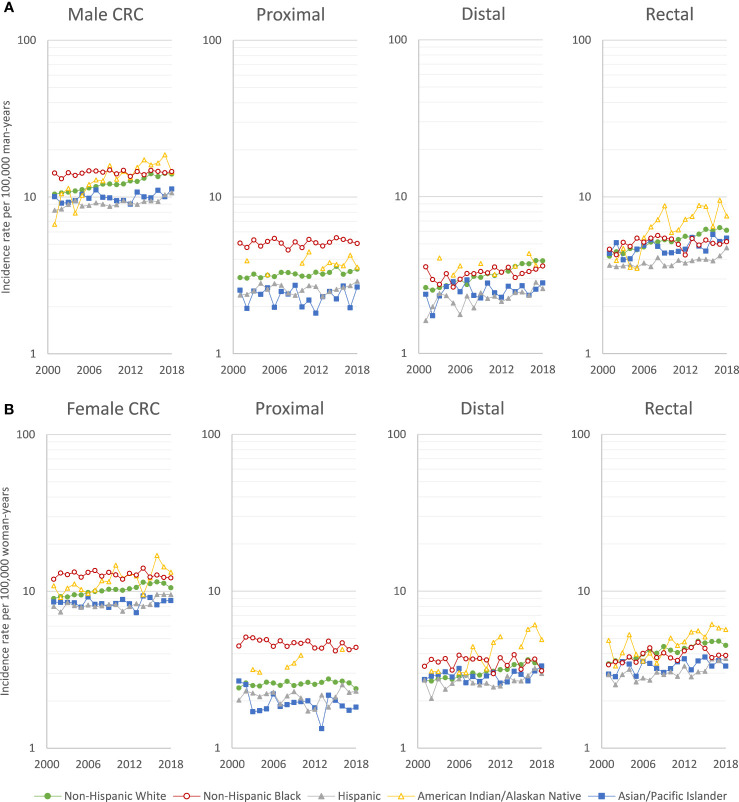
Age-adjusted early-onset (20-49 years of age) colorectal cancer incidence rates per 100,000 person-years in **(A)** men and **(B)** women, US Cancer Statistics 2001-2018.

While overall CRC rates decreased between 2001 and 2018, EOCRC rates increased in NHWs, Hispanics, and AIANs, but remained stable in NHBs and APIs (**Figure 1**). However, early-onset distal colon and rectal cancer rates increased in all racial/ethnic groups. Similar temporal trends in EOCRC rates were observed for men (**Figure 1A**) and women (**Figure 1B**).

EOCRC incidence rates increased by 3.13% per year for AIANs, and by 1.62% per year for NHWs, and by 1.10% per year for Hispanics (**Table 1**), with a more rapid rates increased of 3.61% per year among Hispanics from 2013 to 2018. In NHWs, there were increases in early-onset rectal (AAPC=2.17), distal colon (AAPC=2.16), and proximal colon cancer (AAPC=0.44). In Hispanics, there were comparable increases. In AIANs, increases in rates of early-onset rectal (AAPC=4.04), distal colon (AAPC=3.07), and proximal colon cancer (AAPC=1.47) were greater than for NHWs, while the increases among APIs were smaller. The EOCRC trends were similar in men (**Supplemental Table S2**) and women (**Supplemental Table S3**), with the exception that early-onset proximal colon cancer decreased in NHB women (AAPC=-0.68). In the sensitivity analysis, trends were similar examining individuals aged 20-44 years (**Supplemental Figure S2** and **Supplemental Tables S4**) or <50 years (**Supplemental Table S5**), rather than 20-49 years.

**Table 1:** Age-adjusted early-onset (20-49 years of age) colorectal cancer incidence rates per 100,000 person-years.

Age 20-49 Years	2001-2002					2017-2018					Trend 1	Trend 2	Joinpoint 1	AAPC	AAPC 95% CI
	Cases	Rate	95% CI	IRR	95% CI	Cases	Rate	95% CI	IRR	95% CI	APC (%)	APC (%)		(%)	
***All Colorectal Cancer***															
Non-Hispanic White	17,299	9.82	(9.67, 9.97)	1.00	–	17,732	12.50	(12.32, 12.69)	1.00	–	1.62*			1.62*	(1.41, 1.82)
Non-Hispanic Black	3,794	13.05	(12.63, 13.47)	1.33	(1.28, 1.38)	4,137	13.26	(12.85, 13.67)	1.06	(1.02, 1.10)	0.05			0.05	(-0.24, 0.35)
Hispanic	2,247	8.00	(7.67, 8.34)	0.81	(0.78, 0.85)	4,629	10.02	(9.73, 10.31)	0.80	(0.78, 0.83)	0.28	3.61	2013-2014	1.10*	(0.14, 2.07)
American Indian/Alaskan Native	186	9.32	(8.03, 10.76)	0.95	(0.82, 1.10)	290	15.11	(13.41, 16.95)	1.21	(1.08, 1.36)	3.13*			3.13*	(2.29, 3.98)
Asian/Pacific Islander	1,008	9.03	(8.48, 9.61)	0.92	(0.86, 0.98)	1,701	9.63	(9.18, 10.10)	0.77	(0.73, 0.81)	0.37			0.37	(-0.03, 0.78)
***Proximal Colon Cancer***															
Non-Hispanic White	4,888	2.78	(2.70, 2.86)	1.00	–	4,198	2.95	(2.86, 3.04)	1.00	–	0.44*			0.44*	(0.20, 0.67)
Non-Hispanic Black	1,408	4.85	(4.60, 5.11)	1.75	(1.64, 1.85)	1,462	4.70	(4.46, 4.95)	1.59	(1.50, 1.69)	-0.21			-0.21	(-0.53, 0.10)
Hispanic	641	2.28	(2.10, 2.46)	0.82	(0.75, 0.89)	1,176	2.55	(2.41, 2.70)	0.86	(0.81, 0.92)	-0.57	3.51	2013-2014	0.43	(-0.88, 1.77)
American Indian/Alaskan Native	49	2.45	(1.81, 3.23)	0.88	(0.66, 1.17)	56	2.90	(2.18, 3.76)	0.98	(0.75, 1.28)	1.47			1.47	(-0.11, 3.07)
Asian/Pacific Islander	274	2.43	(2.15, 2.74)	0.88	(0.78, 0.99)	361	2.03	(1.82, 2.25)	0.69	(0.62, 0.77)	-0.57			-0.57	(-1.40, 0.26)
***Distal Colon Cancer***															
Non-Hispanic White	4,654	2.64	(2.56, 2.71)	1.00	–	5,137	3.63	(3.53, 3.73)	1.00	–	2.16*			2.16*	(1.91, 2.41)
Non-Hispanic Black	983	3.38	(3.17, 3.60)	1.28	(1.20, 1.37)	1,075	3.45	(3.25, 3.67)	0.95	(0.89, 1.02)	0.19			0.19	(-0.27, 0.66)
Hispanic	577	2.09	(1.92, 2.27)	0.79	(0.73, 0.86)	1,342	2.91	(2.75, 3.07)	0.80	(0.75, 0.85)	1.43*			1.43*	(0.60, 2.27)
American Indian/Alaskan Native	52	2.60	(1.94, 3.41)	0.99	(0.75, 1.30)	85	4.40	(3.51, 5.44)	1.21	(0.98, 1.50)	3.07*			3.07*	(0.82, 5.37)
Asian/Pacific Islander	269	2.44	(2.16, 2.75)	0.93	(0.82, 1.05)	523	2.97	(2.72, 3.23)	0.82	(0.75, 0.89)	0.47			0.47	(-0.32, 1.27)
***Rectal Cancer***															
Non-Hispanic White	6,910	3.92	(3.83, 4.02)	1.00	–	7,732	5.45	(5.33, 5.58)	1.00	–	2.17*			2.17*	(1.87, 2.46)
Non-Hispanic Black	1,144	3.93	(3.70, 4.16)	1.00	(0.94, 1.07)	1,396	4.45	(4.22, 4.70)	0.82	(0.77, 0.86)	0.58			0.58	(-0.34, 1.50)
Hispanic	896	3.18	(2.97, 3.40)	0.81	(0.76, 0.87)	1,887	4.07	(3.89, 4.26)	0.75	(0.71, 0.79)	0.61	3.98	2013-2014	1.44*	(0.57, 2.32)
American Indian/Alaskan Native	74	3.72	(2.92, 4.67)	0.95	(0.75, 1.19)	135	7.12	(5.96, 8.42)	1.30	(1.10, 1.55)	4.04*			4.04*	(2.87, 5.23)
Asian/Pacific Islander	423	3.77	(3.42, 4.15)	0.96	(0.87, 1.06)	763	4.34	(4.03, 4.65)	0.79	(0.74, 0.86)	0.93*			0.93*	(0.37, 1.50)

*Statistically significant at the 0.05 level.

IRR, incidence rate ratio; CI, confidence interval; APC, annual percent change; AAPC, average annual percent change.

Due to the significant temporal increase in EOCRC rates, AIANs had the highest EOCRC rates in 2017-2018 (ASIR=15.11 per 100,000), followed by NHBs (ASIR=13.26) and NHWs (ASIR=12.50; **Table 1**). The rate of early-onset proximal colon cancer was highest in NHBs (ASIR=4.70), while early-onset distal colon (ASIR=4.40) and rectal cancer (ASIR=7.12) rates were highest in AIANs. The rate of early-onset proximal colon cancer (ASIR=2.03) was lowest in APIs, while early-onset distal colon (ASIR=2.91) and rectal cancer (ASIR=4.07) rates were lowest in Hispanics.

Examining the racial/ethnic disparities in EOCRC, AIANs had a 21% higher incidence of EOCRC than NHWs, while Hispanics and APIs had a 20–23% lower incidence (**Table 1**). Due to increasing EOCRC rates in NHWs, racial disparities in incidence of EOCRC narrowed between NHBs and NHWs (2001-2002 IRR=1.33 to 2017-2018 IRR=1.06). However, NHBs still had a disproportionate burden of early-onset proximal colon cancer in 2017-2018 (IRR=1.59), compared to NHWs.

Rates of late-onset CRC (CRC in individuals aged 50-74 years) decreased during 2001-2018 in all racial/ethnic groups for both sexes (**Supplemental Figure S3**). As shown in **Table 2**, recent declines in late-onset CRC rates among NHWs (2011-2018 APC=-1.41) were less than among NHBs (AAPC=-2.48), Hispanics (AAPC=-1.77), and APIs (AAPC=-1.84), but racial/ethnic disparities in late-onset CRC incidence remain. Rates of late-onset CRC in NHBs were 29% higher and rates in AIANs were 15% higher, compared to NHWs. NHBs had a disproportionate burden of late-onset proximal colon (IRR=1.53), distal colon (IRR=1.22), and rectal cancer (IRR=1.04). AIANs also had a disproportionate burden of late-onset rectal cancer (IRR=1.21). APIs had lower rates of late-onset proximal colon cancer than NHWs (IRR=0.62) but higher rates of distal colon cancer (IRR=1.12). Rates of late-onset CRC were similar between Hispanics and NHWs. These late-onset CRC trends were similar in men (**Supplemental Table S6**) and women (**Supplemental Table S7**). In the sensitivity analysis, trends were similar examining individuals ≥50 years of age (**Supplemental Table S8**), rather than 50-74 years.

**Table 2:** Age-adjusted late-onset (50-74 years of age) colorectal cancer incidence rates per 100,000 person-years.

Age 50-74 Years	2001-2002					2017-2018					Trend 1	Trend 2	Trend 3	Joinpoint 1	Joinpoint 2	AAPC	AAPC 95% CI
	Cases	Rate	95% CI	IRR	95% CI	Cases	Rate	95% CI	IRR	95% CI	APC (%)	APC (%)	APC (%)			(%)	
***All Colorectal Cancer***																	
Non-Hispanic White	121,306	126.08	(125.38, 126.78)	1.00	–	111,516	81.49	(81.00, 81.98)	1.00	–	-3.51*	-1.41*		2011-2012		-2.73*	(-3.10, -2.35)
Non-Hispanic Black	18,401	152.64	(150.38, 154.93)	1.21	(1.19, 1.23)	21,957	105.46	(104.05, 106.88)	1.29	(1.28, 1.31)	-2.48*					-2.48*	(-2.81, -2.15)
Hispanic	9,204	104.72	(102.53, 106.95)	0.83	(0.81, 0.85)	15,903	79.52	(78.27, 80.79)	0.98	(0.96, 0.99)	-1.77*					-1.77*	(-2.11, -1.42)
American Indian/Alaskan Native	754	108.23	(100.50, 116.40)	0.86	(0.80, 0.92)	1,283	94.12	(88.97, 99.49)	1.15	(1.09, 1.22)	-0.62*					-0.62*	(-1.14, -0.10)
Asian/Pacific Islander	4,177	95.28	(92.33, 98.30)	0.76	(0.73, 0.78)	6,800	70.03	(68.36, 71.73)	0.86	(0.84, 0.88)	-1.84*					-1.84*	(-2.02, -1.66)
***Proximal Colon Cancer***																	
Non-Hispanic White	46,241	47.53	(47.09, 47.96)	1.00	–	41,552	29.60	(29.31, 29.89)	1.00	–	-3.06*					-3.06*	(-3.32, -2.79)
Non-Hispanic Black	8,095	65.79	(64.31, 67.31)	1.38	(1.35, 1.42)	9,380	45.36	(44.43, 46.30)	1.53	(1.50, 1.57)	-2.56*					-2.56*	(-2.94, -2.18)
Hispanic	3,209	36.02	(34.73, 37.35)	0.76	(0.73, 0.79)	5,375	27.64	(26.90, 28.41)	0.93	(0.91, 0.96)	-1.78*					-1.78*	(-2.08, -1.48)
American Indian/Alaskan Native	256	35.64	(31.23, 40.48)	0.75	(0.66, 0.85)	419	31.04	(28.10, 34.21)	1.05	(0.95, 1.15)	-0.61					-0.61	(-1.58, 0.37)
Asian/Pacific Islander	1,128	26.54	(24.97, 28.18)	0.56	(0.53, 0.59)	1,757	18.23	(17.38, 19.11)	0.62	(0.59, 0.65)	-2.27*					-2.27*	(-2.65, -1.88)
***Distal Colon Cancer***																	
Non-Hispanic White	32,625	34.11	(33.75, 34.48)	1.00	–	26,647	19.76	(19.52, 20.01)	1.00	–	-4.70*	-1.20		2011-2012		-3.40*	(-4.00, -2.81)
Non-Hispanic Black	4,736	39.84	(38.68, 41.02)	1.17	(1.13, 1.20)	5,060	24.18	(23.51, 24.86)	1.22	(1.19, 1.26)	-1.71	-4.10*	-2.95*	2005-2006	2011-2012	-3.08*	(-3.31, -2.84)
Hispanic	2,531	28.61	(27.47, 29.79)	0.84	(0.81, 0.87)	4,039	20.00	(19.38, 20.64)	1.01	(0.98, 1.05)	-2.43*					-2.43*	(-2.95, -1.90)
American Indian/Alaskan Native	210	29.22	(25.24, 33.63)	0.86	(0.75, 0.98)	314	22.95	(20.45, 25.67)	1.16	(1.04, 1.30)	-1.19*					-1.19*	(-2.09, -0.28)
Asian/Pacific Islander	1,397	31.64	(29.95, 33.39)	0.93	(0.88, 0.98)	2,155	22.07	(21.14, 23.03)	1.12	(1.07, 1.17)	-2.21*					-2.21*	(-2.54, -1.89)
***Rectal Cancer***																	
Non-Hispanic White	37,177	38.32	(37.93, 38.71)	1.00	–	38,135	28.42	(28.13, 28.71)	1.00	–	-2.77*	-0.24		2011-2012		-1.83*	(-2.05, -1.62)
Non-Hispanic Black	4,445	36.61	(35.51, 37.73)	0.96	(0.93, 0.99)	6,218	29.69	(28.94, 30.45)	1.04	(1.02, 1.07)	-1.39*					-1.39*	(-1.75, -1.03)
Hispanic	3,014	34.30	(33.06, 35.58)	0.90	(0.86, 0.93)	5,661	27.66	(26.93, 28.40)	0.97	(0.95, 1.00)	-1.23*					-1.23*	(-1.66, -0.79)
American Indian/Alaskan Native	250	36.66	(32.27, 41.47)	0.96	(0.84, 1.08)	472	34.39	(31.31, 37.68)	1.21	(1.10, 1.32)	-0.12					-0.12	(-0.80, 0.56)
Asian/Pacific Islander	1,511	33.91	(32.17, 35.71)	0.88	(0.84, 0.93)	2,663	27.42	(26.38, 28.49)	0.96	(0.93, 1.00)	-1.20*					-1.20*	(-1.43, -0.97)

*Statistically significant at the 0.05 level.

IRR, incidence rate ratio; CI, confidence interval; APC, annual percent change; AAPC, average annual percent change.

In **Tables 3**, **4**, rates of early- and late-onset CRC are shown by histology: colorectal adenocarcinoma accounted for 90.9% of all CRCs, while colorectal neuroendocrine tumors accounted for 4.1%. The majority (73.7%) of colorectal neuroendocrine tumors are located in the rectum; thus, only overall CRC rates are provided by histology. The rates and trends for CRC and colorectal adenocarcinoma were very similar (**Table 3**). The incidence of colorectal neuroendocrine tumors was about one-tenth of that of colorectal adenocarcinoma; for example, NHB rates of early-onset colorectal adenocarcinoma in 2017-2018 were 11.13 per 100,000 (**Table 3**), while rates of early-onset colorectal neuroendocrine tumors were 1.53 per 100,000 (**Table 4**). As shown in **Table 4**, rates of early-onset colorectal neuroendocrine tumors increased from 2001 to 2018 in NHWs by 2.22%, in NHBs by 1.97%, and in Hispanics by 1.87% per year. Rates of late-onset colorectal neuroendocrine tumors increased in NHBs by 1.95%, in Hispanics by 2.17%, in AIANs by 3.04%, and in APIs by 3.24% per year. The racial/ethnic disparities were more pronounced for colorectal neuroendocrine tumors (compared to colorectal adenocarcinoma), particularly for NHBs compared to NHWs. In 2017-2018, incidence was 2.35-times higher for early-onset and 3.26-times higher for late-onset colorectal neuroendocrine tumors in NHBs, compared to NHWs.

**Table 3:** Age‐adjusted early‐onset (20-49 years of age) and late‐onset (50-74 years of age) colorectal adenocarcinoma rates per 100,000 person‐years.

All Colorectal Cancer	2001-2002					2017-2018					Trend 1	Trend 2	Joinpoint 1	AAPC	AAPC 95% CI
	Cases	Rate	95% CI	IRR	95% CI	Cases	Rate	95% CI	IRR	95% CI	APC (%)	APC (%)		(%)	
***20-49 Years***															
Non-Hispanic White	15,641	8.87	(8.73, 9.01)	1.00	–	16,093	11.36	(11.18, 11.53)	1.00	–	1.69*			1.69*	(1.50, 1.89)
Non-Hispanic Black	3,273	11.26	(10.88, 11.65)	1.27	(1.22, 1.32)	3,465	11.13	(10.76, 11.51)	0.98	(0.94, 1.02)	-0.02			-0.02	(-0.30, 0.25)
Hispanic	1,962	7.01	(6.70, 7.34)	0.79	(0.75, 0.83)	4,048	8.78	(8.51, 9.05)	0.77	(0.75, 0.80)	1.05*			1.05*	(0.43, 1.68)
American Indian/Alaskan Native	166	8.33	(7.11, 9.70)	0.94	(0.81, 1.09)	258	13.42	(11.82, 15.16)	1.18	(1.04, 1.34)	3.07*			3.07*	(2.31, 3.83)
Asian/Pacific Islander	888	7.96	(7.44, 8.50)	0.90	(0.84, 0.96)	1,502	8.51	(8.09, 8.96)	0.75	(0.71, 0.79)	0.35			0.35	(-0.06, 0.75)
***50-74 Years***															
Non-Hispanic White	115,547	118.15	(117.47, 118.84)	1.00	–	101,799	74.26	(73.80, 74.73)	1.00	–	-3.72*	1.54*	2011-2012	-2.91*	(-3.28, -2.53)
Non-Hispanic Black	15,905	138.59	(136.43, 140.77)	1.17	(1.15, 1.19)	18,810	90.32	(89.02, 91.64)	1.22	(1.20, 1.24)	-2.82*			-2.82*	(-3.13, -2.51)
Hispanic	8,109	96.14	(94.04, 98.28)	0.81	(0.80, 0.83)	14,163	71.02	(69.83, 72.22)	0.96	(0.94, 0.97)	-1.97*			-1.97*	(-2.33, -1.61)
American Indian/Alaskan Native	692	98.44	(91.05, 106.25)	0.83	(0.77, 0.90)	1,170	85.75	(80.84, 90.88)	1.15	(1.09, 1.22)	-0.74*			-0.74*	(-1.17, -0.30)
Asian/Pacific Islander	3,727	88.30	(85.46, 91.21)	0.75	(0.72, 0.77)	6,050	62.24	(60.66, 63.84)	0.84	(0.82, 0.86)	-2.12*			-2.12*	(-2.26, -1.98)

*Statistically significant at the 0.05 level.

IRR, incidence rate ratio; CI, confidence interval; APC, annual percent change; AAPC, average annual percent change.

**Table 4:** Age‐adjusted early‐onset (20-49 years of age) and late‐onset (50-74 years of age) colorectal neuroendocrine rates per 100,000 person‐years.

All Colorectal Cancer	2001-2002					2017-2018					Trend 1	Trend 2	Joinpoint 1	AAPC	AAPC 95% CI
	Cases	Rate	95% CI	IRR	95% CI	Cases	Rate	95% CI	IRR	95% CI	APC (%)	APC (%)		(%)	
***20-49 Years***															
Non-Hispanic White	755	0.43	(0.40, 0.47)	1.00	–	924	0.65	(0.61, 0.69)	1.00	–	2.22*			2.22*	(1.50, 2.94)
Non-Hispanic Black	272	0.93	(0.82, 1.05)	2.14	(1.86, 2.46)	480	1.53	(1.39, 1.67)	2.35	(2.11, 2.63)	1.97*			1.97*	(0.45, 3.51)
Hispanic	129	0.44	(0.37, 0.53)	1.02	(0.85, 1.23)	330	0.70	(0.63, 0.78)	1.08	(0.95, 1.23)	1.87*			1.87*	(0.22, 3.54)
American Indian/Alaskan Native	–					21	1.13	(0.70, 1.73)	1.75	(1.13, 2.69)	–			–	
Asian/Pacific Islander	78	0.70	(0.55, 0.87)	1.61	(1.28, 2.04)	139	0.78	(0.66, 0.92)	1.20	(1.01, 1.44)	0.79			0.79	(-0.96, 2.56)
***50-74 Years***															
Non-Hispanic White	2,318	2.36	(2.27, 2.46)	1.00	–	3,634	2.87	(2.77, 2.97)	1.00	–	0.79			0.79	(-0.21, 1.79)
Non-Hispanic Black	735	6.12	(5.68, 6.58)	2.59	(2.38, 2.81)	1,949	9.36	(8.95, 9.79)	3.26	(3.09, 3.45)	1.95*			1.95*	(0.84, 3.07)
Hispanic	262	2.92	(2.57, 3.30)	1.24	(1.09, 1.40)	870	4.11	(3.84, 4.40)	1.43	(1.33, 1.54)	2.17*			2.17*	(1.01, 3.33)
American Indian/Alaskan Native	22	2.67	(1.66, 4.09)	1.13	(0.74, 1.72)	47	3.53	(2.58, 4.70)	1.23	(0.92, 1.64)	3.04*			3.04*	(0.83, 5.30)
Asian/Pacific Islander	154	3.41	(2.89, 4.00)	1.44	(1.23, 1.70)	483	5.05	(4.61, 5.53)	1.76	(1.60, 1.94)	8.67	0.11	2007-2008	3.24*	(0.35, 6.21)

*Statistically significant at the 0.05 level.

IRR, incidence rate ratio; CI, confidence interval; APC, annual percent change; AAPC, average annual percent change.

**Figure 2:**
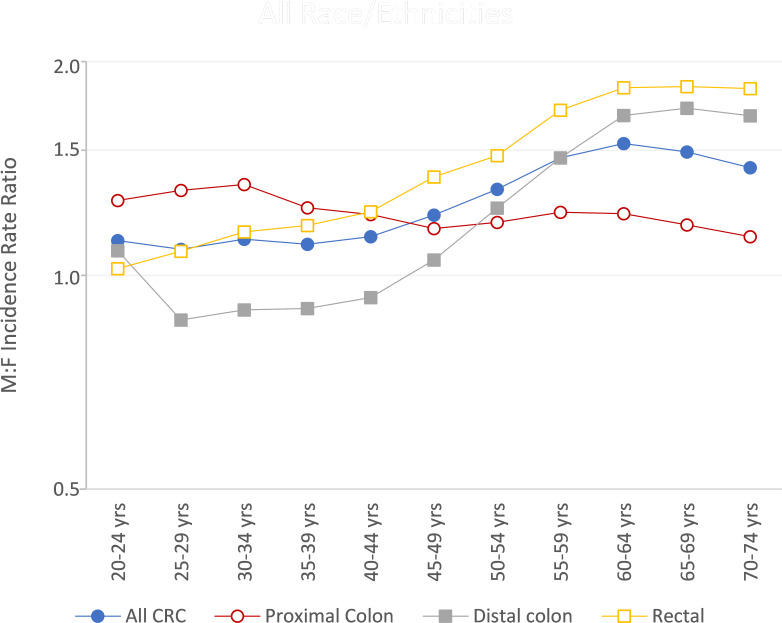
Colorectal cancer male-to-female (M/F) incidence rate ratios by age and subsite, US Cancer Statistics 2001-2018.

Sex differences were observed for both early- and late-onset CRC, with men having 16% higher rates of EOCRC and 44% higher rates of late-onset CRC (**Supplemental Figure S4**). At all ages, rates of CRC were higher in men than women for proximal colon and rectal cancer (**Figure 2**). For the ages of 25 to 44, however, women had higher rates of distal colon cancer than men. This sex difference in early-onset distal colon cancer incidence was present in every racial/ethnic group (**Supplemental Figure S5**). In men, the largest proportion of CRCs were rectal for both early- and late-onset disease (42.3% and 34.5%, respectively, data not shown). The exception was NHB men, who had equal proportions of EOCRC occurring in the rectum (35.7%) and proximal colon (35.7%); NHB men had highest proportion of late-onset CRC occurring in the proximal colon (39.6%). For women, the largest proportion of EOCRCs were rectal (38.7%), with the exception of NHBs who had the highest proportion of CRC occurring in the proximal colon (36.1%). The largest proportion of late-onset CRCs in women were proximal (42.4%), with the exception of APIs who had the highest proportion of CRC occurring in the rectum (34.3%).

## Discussion

In recent years, incidence rates of EOCRC increased for NHWs, Hispanics, and AIANs. Incidence of EOCRC in AIANs increased by an average of 3.13% per year from 2001 to 2018. AIANs now have the highest rate of EOCRC, which is 21% higher than incidence of EOCRC in NHWs. NHBs have the second highest incidence of EOCRC; NHBs the highest incidence of late-onset CRC, which is 29% higher than incidence of late-onset CRC in NHWs. Rates of early- and late-onset colorectal neuroendocrine tumors are increasing in all racial/ethnic groups. Men have higher rates of CRC than women, with the exception of early-onset distal colon cancer.

The current study updates and expands recent US reports (1, 2, 16, 17), through interrogation of CRC rates by anatomic subsite, considering age, race/ethnicity, sex, and histology. A recent study, based on data from 1992-2014, reported increasing rates of early-onset distal colon and rectal cancer in NHWs and to a lesser extent NHBs. For early-onset proximal colon cancer, the prior study identified increasing rates for NHWs but decreasing rates for NHBs (17). Similarly, we report that increasing EOCRC rates in NHWs were driven predominately by large increases in distal colon and rectal cancer. However, we also report rates of early-onset distal colon cancer have increased in Hispanics and AIANs, while rates of early-onset rectal cancer have increased in Hispanics, AIANs, and APIs. Additionally, we found that in recent years early-onset proximal cancer increased among NHWs, Hispanics, and AIANs. We did not find that rates of any EOCRC were increasing in NHBs. Similar to the prior report (17), rates of early-onset proximal cancer have decreased in NHBs, specifically in NHB women. Another recent report, based on data from 1995-2016, reported that the increasing rates of EOCRC in NHWs resulted in recent EOCRC being equivalent between NHWs and NHBs (2). We found that EOCRC rates were similar between NHWs and NHBs, but EOCRC rates remain higher in NHBs.

Risk factors for late-onset colorectal adenocarcinoma, which accounts for the majority of CRC, are well researched including obesity (7–9), physical inactivity (10), smoking (8, 11), alcohol (12), and diet (12). The few studies of colorectal neuroendocrine tumors (24–26) raised the possibility that alcohol, metabolic syndrome, and cholesterol levels may be associated with an increased risk. A recent report, using the SEER Program database, reported that rates of neuroendocrine tumors, including colorectal, have increased (27), and that neuroendocrine tumors were more likely to occur in non-White racial/ethnic groups (27), especially distant-stage gastrointestinal neuroendocrine tumors. Another study, also based in the SEER Program database, reported that early-onset colorectal neuroendocrine tumors have increased more rapidly than early-onset adenocarcinomas (28), but did not consider these trends by race/ethnicity or for late-onset CRC. We found that rates of early-onset colorectal neuroendocrine tumors are rapidly increasing, including in NHBs for whom rates of early-onset colorectal adenocarcinoma are not increasing. We also found that rates of late-onset colorectal neuroendocrine tumors are increasing, while rates of late-onset colorectal adenocarcinomas are decreasing in all racial/ethnic groups.

One postulated risk factor for EOCRC is the gut microbiota, which has complex, multifactorial influences (e.g., diet, pathogens, stress, medications, tobacco/alcohol use, physical activity, genetics) (29) and has been reported to vary by race/ethnicity (30) and sex (31). Additionally, gut microbiome profiles have been reported to differ by CRC molecular subtype (32), which differ by anatomic location (33). A recent study supported the influence of the microbiota on EOCRC, reporting that the microbiome within tumors arising before age 45 were more likely to include *Fusobacterium nucleatum*, which promotes colorectal tumorigenesis in the tumor microenvironment by suppressing the immune response, and less likely to include *Moraxella osloensis* than tumors arising after age 65 (34). Identification of microbial profiles specific to EOCRC may yield insights into the etiology of EOCRC and inform novel therapeutics and cancer screening strategies (34).

Historically, NHBs have had the highest rates of CRC in the US, including EOCRC (1, 17). We found that AIANs now have the highest rates of EOCRC and the second highest rate of late-onset CRC. AIANs have long had high rates of CRC, with the highest CRC rates in the US reported among ANs (35, 36). This could be in part due to higher prevalence of risk factors in the AIAN population (e.g., poor diet, vitamin D deficiency, smoking, obesity, diabetes, and *Helicobacter pylori* infection) (36–40), but it is likely that discrimination, historic healthcare administration policies, and structural challenges also play a major role (41). Perceived racial/ethnic medical discrimination has been linked with lower receipt of preventive cancer screening, including CRC screening (42), and nearly a quarter of AIANs report experiencing discrimination when accessing healthcare (43). Regardless of whether AIANs have other health insurance, including Medicare, the Indian Health Service is the primary federal agency that fulfills the US government responsibility to provide healthcare services to AIANs (44, 45). The Indian Health Service spends approximately half the amount per capita on healthcare for AIANs compared to per capita expenditures for federal inmates or Medicaid recipients (41). In 2016, the Indian Health Service reported that less than 40% of screening aged individuals had received appropriate CRC screening (i.e., stool testing in the past year, using a fecal occult blood test [FOBT] or a fecal immunochemical test [FIT]; flexible sigmoidoscopy in the past 5 years; or colonoscopy in the past 10 years) (46). The lack of screening for and removal of adenomas, the precursors to most colorectal adenocarcinomas, could result in higher incidence rates of late-onset CRC. Due to the logistics of endoscopy locations and resource availability, the primary mode of CRC screening in the Indian Health Service tends to be stool testing. Current guidelines from the US Multi-Society Task Force of Colorectal Cancer recommend colonoscopy every 10 years or FIT annually as the first-tier CRC screening tests (47). FIT tests are more acceptable than colonoscopy to most individuals (48), but adherence to an annual regimen and lower endoscopic follow-up for abnormal results can be challenging—especially for ANs, many of whom live in remote areas where access to endoscopy can require long-distance, high-cost air travel (49, 50). Further, the Indian Health Service is not health insurance. If endoscopy is not available at an Indian Health Service or tribal facility, it can be purchased through Contract Health Services. However, a lower endoscopy is not considered a high priority referral and could be denied, potentially impacting rates of both early- and late-onset CRC (45). Current strategies to increase screening in the AIAN population include close partnerships with the community to distribute culturally-sensitive information on CRC screening and implementation of patient navigation and provider education (49, 51, 52). Specific projects have trained mid-level healthcare providers (e.g., physician assistants and nurse practitioners) in rural areas to provide lower endoscopy (49) and mailed FIT kits to individuals with diabetes or pre-diabetes (51).

NHBs have the second highest rate of EOCRC and the highest rate of late-onset CRC. While the racial disparity between NHBs and NHWs in incidence of EOCRC has narrowed, this is due to increasing rates in NHWs, not to decreasing rates in NHBs. Due to the large increases in the overall EOCRC rate (primarily driven by the increases in rates of colorectal adenocarcinoma in NHWs), there is intense interest in understanding EOCRC etiology. In addition to the microbiome, discussed above, obesity is a primary risk factor of interest for EOCRC. Recent literature has shown conflicting results for an obesity–EOCRC association, with a study from a primarily NHW population showing an obesity is associated with an increased EOCRC risk (9) and a study from a primarily NHB population showing no association (53). Thus, obesity may not explain the high rates of EOCRC in NHBs. Another potential risk factor that has received little attention to date is increased levels of stress in younger birth cohorts (13, 54). One study has reported that perceived stress is associated with an increased risk of CRC (55), potentially through stress inducing genetic, epigenetic, microbial, and immune alterations (13). If stress increases EOCRC risk, this could disproportionately affect NHBs, compared to NHWs, as NHBs face additional stressors due to systemic, institutional, and individual racism. NHBs report experiencing the highest levels of both general racism (56%) and health care racism (13%) (56).

Disparities in CRC incidence between NHBs and NHWs persist after adjusting for risk factors and socioeconomic status (57). Further, in populations where the access to care is expected to be similar between racial/ethnic groups (e.g., active-duty military or veterans), the disparities in CRC incidence are even more pronounced (58–62). Thus, high rates of late-onset CRC in NHBs may be driven by social determinants of health and structural racism, which together with historic abuse and exploitation of NHB individuals (63) can hinder receipt of preventive screenings (64, 65). A recent study demonstrated that when NHB patients received care from NHB physicians, patients received more preventive services and had more trust in the healthcare system and patient-provider communication increased (65). The proportion of physicians who are NHB does not reflect the proportion of NHBs in the US population (66). Thus, pipeline programs for under-represented minorities to enter into healthcare careers are necessary to create a healthcare workforce that reflects the population it serves (64, 67). In addition, all physicians should receive training in implicit bias, cultural competency, and patient centeredness and practice in operationalizing these skills to improve communication with patients of different racial/ethnic groups (64, 68, 69).

Across all racial/ethnic groups, men have higher rates of CRC, and the sex differences in rates increase with age. In 2018, men and women had nearly equivalent rates of CRC screening (2). Thus, the higher rates of CRC in men are primarily attributed to modifiable risk factors that differ by sex—diets high in red meat, alcohol consumption, smoking, and visceral adiposity (70). However, the sex difference noted in the screening-aged population could also be due, in part, to some men reporting embarrassment and offense with CRC screening, which healthcare providers should be cognizant of when promoting CRC screening to men (71, 72).

In women, proximal colon cancer is the predominate type of late-onset CRC. Proximal colon cancers are more likely than distal colon or rectal cancer to have high microsatellite instability (MSI) (33, 73, 74), which is hypothesized to be partially due to high estrogen levels. A recent study demonstrated that proxies of higher estrogen levels, including pregnancy, oral contraceptive use, and menopausal hormone therapy use, were associated with lower risk of MSI-high CRC in women (75). Thus, the predominance of late-onset proximal colon cancer in women may be partially due to declining estrogen levels. Proximal and distal colon cancer have different embryologic origins—the embryonic midgut and hindgut, respectively, which may also partially explain the molecularly distinct profiles and presentation of these cancer types (74).

The new ACS and USPSTF guidelines recommend screening beginning at age 45 (5, 6). However, rates of EOCRC are continuing to increase in individuals under age 45. Thus, additional research should focus on underlying causes and identifying predictors of EOCRC to determine if certain groups should be screened prior to age 45. For individuals aged 45 to 50 years, consistent messaging and access to healthcare are needed to ensure that these individuals obtain CRC screening, especially in AIAN and NHB communities. Flexible sigmoidoscopy, which only evaluates the distal colon and rectum, has been suggested as a screening tool for EOCRC (14, 76). However, proximal colon cancer is the predominant form of both early- and late-onset CRC in NHB men and women. Thus, NHBs should ideally receive a colonoscopy screening to fully evaluate the proximal colon.

The strength of this study is use of the US Cancer Statistics database (covering 99% of the US population) versus the more commonly used SEER Program database (covering up to 35% of the US population). Use of high-quality population-based cancer registry data from the entire US population allowed us to comprehensively explore racial/ethnic disparities and sex differences. Hispanic ethnicity may have been incorrectly classified for some individuals, as the US Cancer Statistics database uses the North American Association of Central Cancer Registries Hispanic Identification Algorithm (77). However, this algorithm has been shown to have high sensitivity (92.9), specificity (98.0), and positive predictive values (95.6) (78). We did not adjust for delayed data reporting, beyond the standard 2-year delay. However, delay in case reporting has been shown to be minimal for CRC, with 97% of cases reported using the standard 2-year delay (79). In addition, there is underreporting due to lack of data from Veterans Affairs hospital patients during some years, but the impact on CRC rates is minimal (80). Finally, a limitation of all cancer registry data is that no information is available on individual risk factors, including genetic predisposition to CRC which is particularly relevant for EOCRC. Thus, we were unable to adjust for individual-level factors.

Future research should focus on identifying risk factors and predictors of EOCRC to determine if there are high-risk groups that should be targeted for screening prior to age 45. Additional research is also needed to determine the etiology of colorectal neuroendocrine tumors, as rates of both early- and late-onset are increasing. Recent increases in EOCRC, primarily driven by increasing rates in NHWs, have elicited intense interest in understanding the underlying etiology of EOCRC. However, studies examining EOCRC etiology need to make certain that racial/ethnic minorities are included, to ensure that these studies can be used to mitigate racial/ethnic disparities in EOCRC. Ongoing prevention efforts must ensure access to appropriate CRC screening for AIANs and NHBs.

## Data Availability Statement

Publicly available datasets were analyzed in this study. This data can be found here: https://www.cdc.gov/cancer/uscs/public-use/obtain-data.htm.

## Author Contributions

JLP participated in the conception, design, and analysis of the study; and drafted the manuscript. LEB participated in the conception, design, and analysis of the study; and provided critical revision of the manuscript for important intellectual content. SWA participated in the design of the study and provided critical revision of the manuscript for important intellectual content. AAF participated in the design of the study and provided critical revision of the manuscript for important intellectual content. JRP participated in the design and analysis of the study and provided critical revision of the manuscript for important intellectual content. LR participated in the conception and design of the study, assisted in drafting the manuscript, and providing revisions of important intellectual content. All authors contributed to the article and approved the submitted version.

## Funding

Karin Grunebaum Cancer Research Foundation (JLP and JRP), Boston University Peter Paul Career Development Professorship (JLP), and National Cancer Institute R00 CA207848 (SWA).

## Conflict of Interest

The authors declare that the research was conducted in the absence of any commercial or financial relationships that could be construed as a potential conflict of interest.

## Publisher’s Note

All claims expressed in this article are solely those of the authors and do not necessarily represent those of their affiliated organizations, or those of the publisher, the editors and the reviewers. Any product that may be evaluated in this article, or claim that may be made by its manufacturer, is not guaranteed or endorsed by the publisher.

## Abbreviations

ASIR, age-standardized incidence rate; ACS, American Cancer Society; AIAN, American Indian/Alaskan Native; APC, annual percent change; API, Asian/Pacific Islander; AAPC, average annual percent change; CRC, colorectal cancer; CIs, confidence intervals; EOCRC, early-onset colorectal cancer; IRR, incidence rate ratios; NPCR, National Program of Cancer Registries; NHB, non-Hispanic Black; NHW, non-Hispanic White; SEER, Surveillance, Epidemiology, and End Results; US, United States; USPSTF, US Preventive Services Task Force.
