# Supplementary material for: Racial Disparities and Sex Differences in Early- and Late-Onset Colorectal Cancer Incidence, 2001–2018
Source: Front Oncol. 2021 Sep 9;11:734998. doi: 10.3389/fonc.2021.734998 (PMC8459723; doi:10.3389/fonc.2021.734998)
Supplement: Supplementary file 1 [file Image_1.pdf]

Supplemental Figure S1. Age-adjusted colorectal cancer incidence rates for all ages per 100,000 person-years in A) men and B) women, US Cancer Statistics 2001-2018.

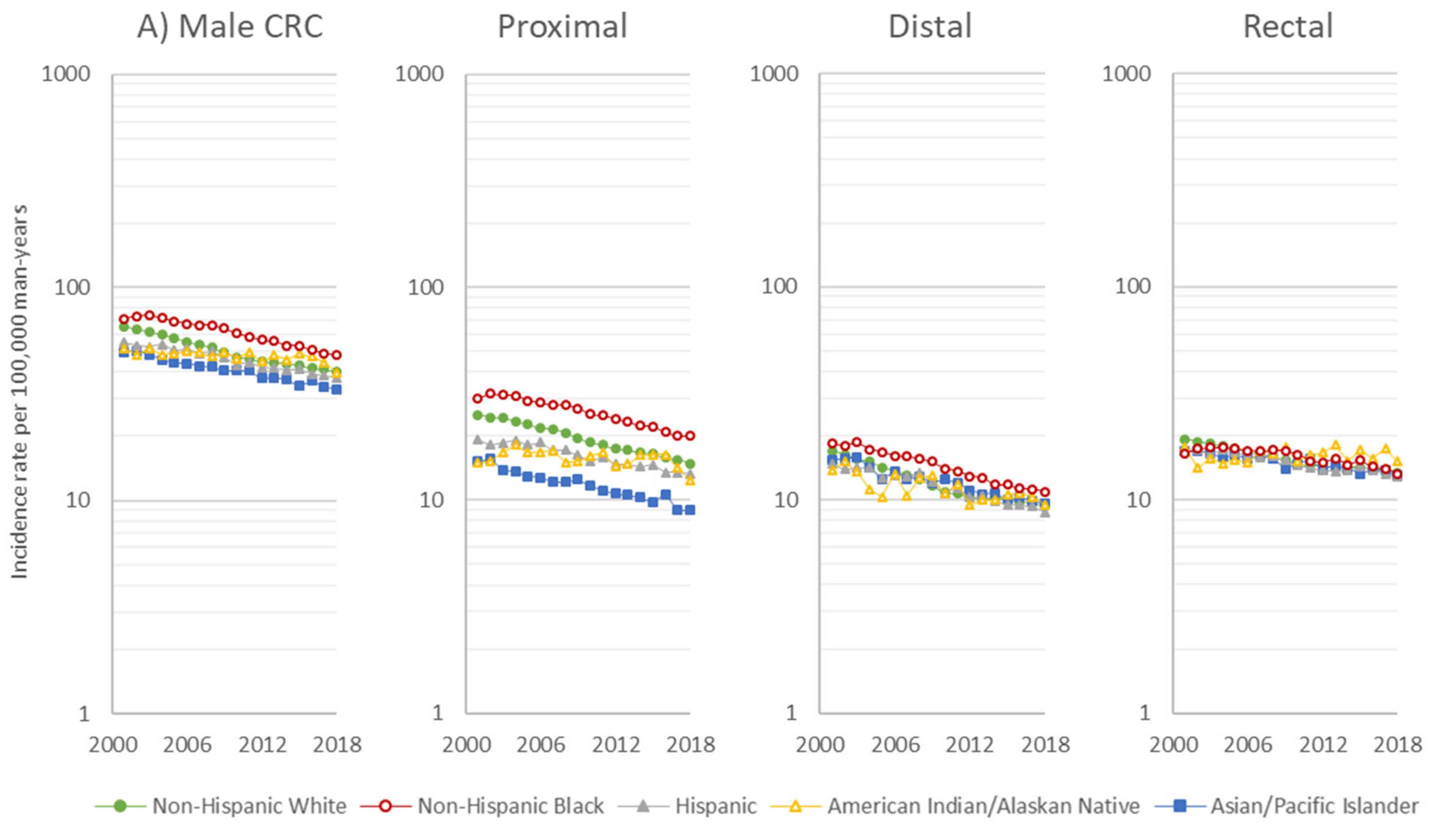

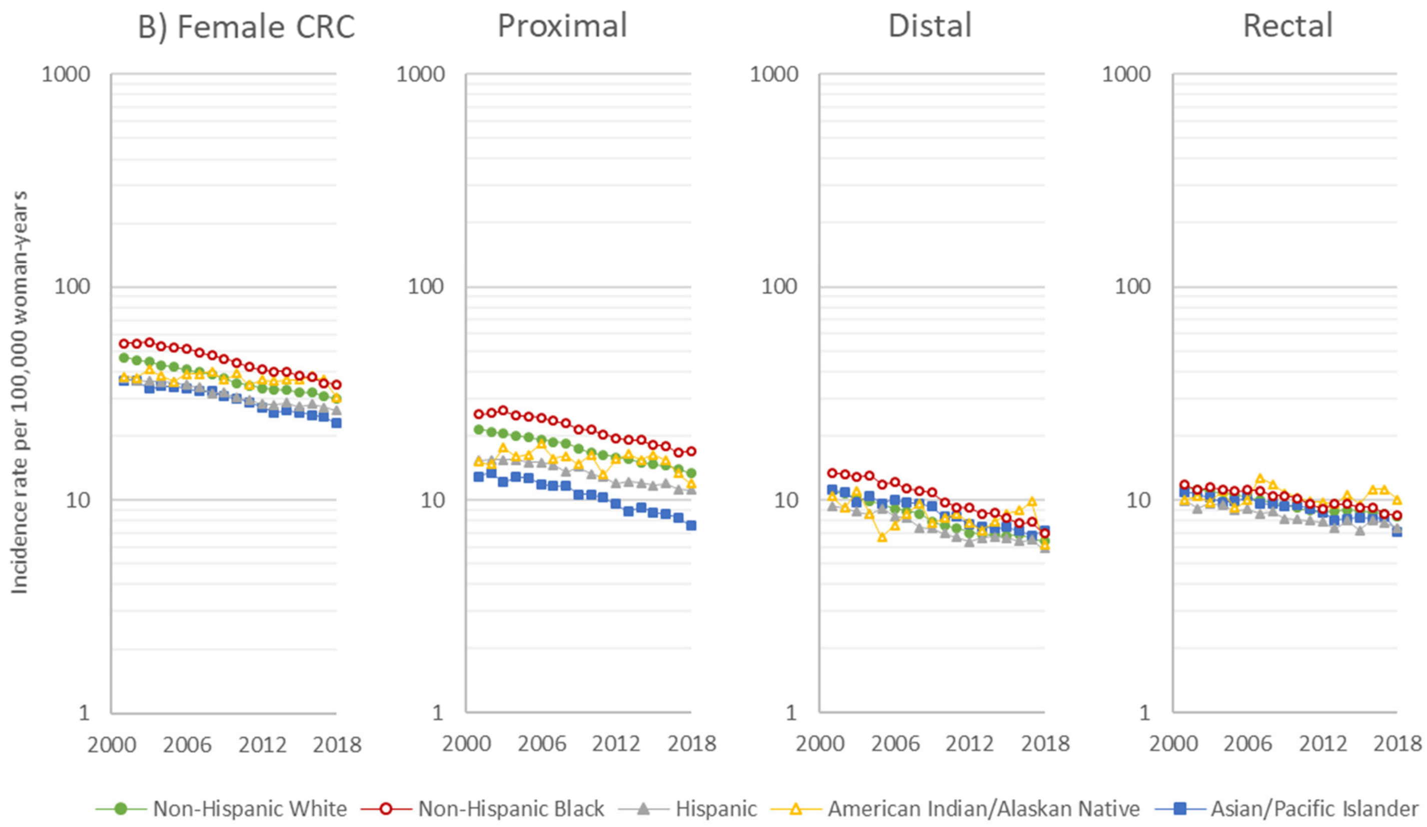
B) Female CRC
Proximal
Distal
Rectal
Incidence rate per 100,000 woman-years
1000
100
10
1
2000
2006
2012
2018
Non-Hispanic White
Non-Hispanic Black
Hispanic
American Indian/Alaskan Native
Asian/Pacific Islander
