# Supplementary material for: Racial Disparities and Sex Differences in Early- and Late-Onset Colorectal Cancer Incidence, 2001–2018
Source: Front Oncol. 2021 Sep 9;11:734998. doi: 10.3389/fonc.2021.734998 (PMC8459723; doi:10.3389/fonc.2021.734998)

Supplemental Figure S2. Age-adjusted early-onset (20-44 years of age) colorectal cancer incidence rates per 100,000 person-years, US Cancer Statistics 2001-2018.

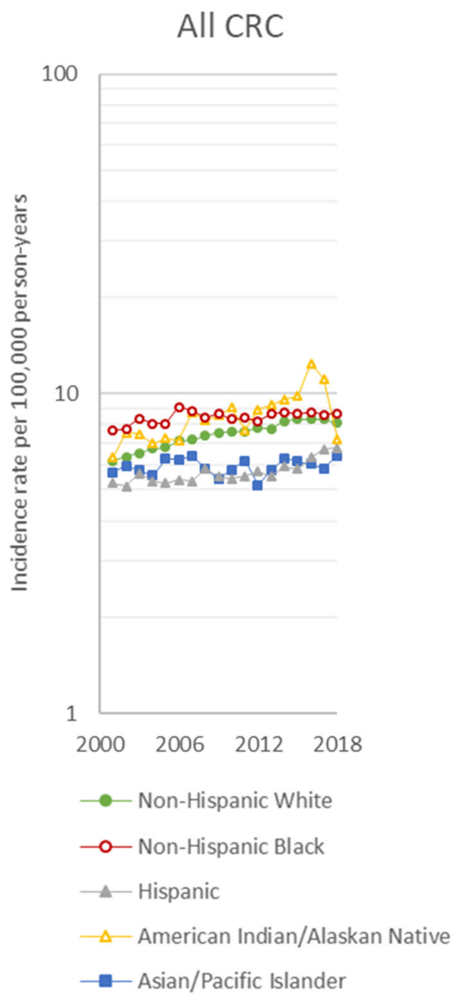

Supplement: Supplementary file 2 [file Image_2.pdf]
