# Supplementary material for: Racial Disparities and Sex Differences in Early- and Late-Onset Colorectal Cancer Incidence, 2001–2018
Source: Front Oncol. 2021 Sep 9;11:734998. doi: 10.3389/fonc.2021.734998 (PMC8459723; doi:10.3389/fonc.2021.734998)

Supplemental Figure S4. Colorectal cancer incidence rates and male-to-female (M/F) incidence rate ratios by A) early- and B) late-onset and subsite, US Cancer Statistics 2001-2018.

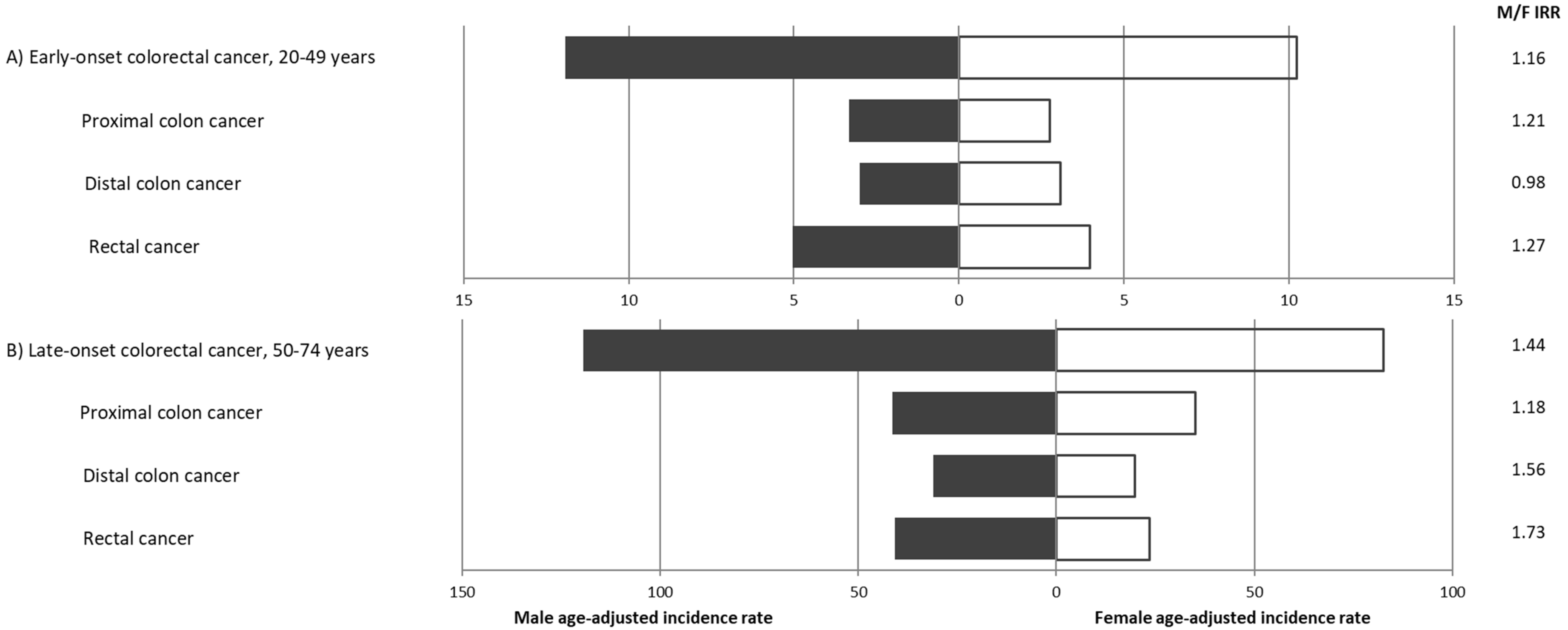

Supplement: Supplementary file 4 [file Image_4.pdf]
